# Health and economic outcomes of 20-valent pneumococcal conjugate vaccine compared to 15-valent pneumococcal conjugate vaccine strategies for adults in Greece

**DOI:** 10.3389/fpubh.2023.1229524

**Published:** 2023-09-29

**Authors:** George Gourzoulidis, Myrto Barmpouni, Vasiliki Kossyvaki, Jeffrey Vietri, Charalampos Tzanetakos

**Affiliations:** ^1^Health Through Evidence, Athens, Greece; ^2^Pfizer Hellas, Athens, Greece; ^3^Pfizer Inc., Collegeville, PA, United States

**Keywords:** PCV20, PCV15, pneumococcal disease, Greece, pneumococcal conjugate vaccines

## Abstract

**Objective:**

Higher valency pneumococcal conjugate vaccines (PCVs) are expected to improve protection against pneumococcal disease through coverage of additional serotypes. The aim of the present study was to evaluate the cost-effectiveness of 20-valent pneumococcal conjugate vaccine (PCV20) compared to 15-valent pneumococcal conjugate vaccine (PCV15) alone or followed by 23-valent polysaccharide vaccine (PPV23) for adults in Greece.

**Methods:**

A published Markov model was adapted to simulate lifetime risk of clinical and economic outcomes from the public payer’s perspective. The model population was stratified based on age and risk profile (i.e., low, moderate, or high-risk of developing pneumococcal disease). Epidemiologic parameters, serotype coverage and vaccines’ effectiveness were based on published literature, while direct medical costs (prices €, 2022) were obtained from official sources. Main model outcomes were projected number of invasive pneumococcal disease (IPD) and all-cause non-bacteremic pneumonia (NBP) cases and attributable deaths, costs and quality-adjusted life-years (QALY) for each vaccination strategy. Sensitivity analyses were performed to ascertain the robustness of model results.

**Results:**

Over the modeled time horizon, vaccination with PCV20 compared to PCV15 alone or PCV15 followed by PPV23 prevents an additional 747 and 646 cases of IPD, 10,334 and 10,342 cases of NBP and 468 and 455 deaths respectively, resulting in incremental gain of 1,594 and 1,536 QALYs and cost savings of €11,183 and €48,858, respectively. PSA revealed that the probability of PCV20 being cost-effective at the predetermined threshold of €34,000 per QALY gained was 100% compared to either PCV15 alone or the combination of PCV15 followed by PPV23.

**Conclusion:**

PCV20 is estimated to improve public health by averting additional pneumococcal disease cases and deaths relative to PCV15 alone or followed by PPV23, and therefore translates to cost-savings for the public payer. Overall results showed that vaccination with PCV20 was estimated to be a dominant vaccination strategy (improved health outcomes with reduced costs) over PCV15 alone or followed by PPV23 for prevention of pneumococcal disease in adults in Greece.

## 1. Introduction

*Streptococcus pneumoniae* can produce a range of infections collectively termed pneumococcal disease (1). Pneumococcal disease is a significant cause of morbidity and mortality globally, especially in patients with comorbidities and advanced age (2). Moreover, pneumococcal disease can be classified as either invasive pneumococcal disease (IPD) or non-invasive pneumococcal disease (3). More specifically, IPDs include infections such as bacteremia/sepsis, meningitis, or bacteremic pneumonia and non-invasive pneumococcal diseases include infections such as non-bacteremic pneumonia, otitis media, or sinusitis.

Non-bacteremic (non-invasive) pneumococcal pneumonia is much more common in adults than IPD, making up approximately 75% of cases of pneumococcal pneumonia (4, 5). Moreover, World Health Organization (WHO) reports that in most countries, pneumococcal surveillance systems are based on invasive disease infections (6), and therefore the burden of pneumococcal disease is most likely underestimated. In 2018, 42 confirmed cases of IPD were reported in Greece and the crude notification rate was 0.4 cases per 100,000 population (7). It has to be noted however that IPD is not under mandatory surveillance in Greece, with the exception of meningitis, therefore this number is probably an underestimate of the actual IPD cases (8).

The implementation of vaccination aimed to reduce the burden of the disease. In Greece, up to the end of 2022 there were 2 types of pneumococcal vaccines available for adults, the 13-valent pneumococcal conjugate vaccine (PCV13), which includes 13 serotypes, and the 23-valent pneumococcal polysaccharide vaccine (PPV23), which includes 23 serotypes. PCV13 was introduced in the adult National Immunization Program (NIP) for all persons ≥50 years of age as of December 2011. Prior to December 2011, Greece lacked a formalized adult NIP, but PPV23, which has been available on the market since 1999, was suggested in individuals deemed to have a higher susceptibility to pneumococcal disease by their attending physicians, although with low vaccination rates. In January 2015, the Greek NIP underwent revision, recommending the administration of PCV13 followed by PPV23 to individuals aged ≥65 years and those aged 19–64 years who are deemed to have an elevated risk of pneumococcal disease (8).

The recent approval of two new pneumococcal conjugate vaccines, 20-valent pneumococcal conjugate vaccine (PCV20) (9) and 15-valent pneumococcal conjugate vaccine (PCV15) (10), for use in Europe and the USA has resulted in an update of immunization program recommendations in European countries (11–14) and the USA (15, 16) in 2022. In addition to the PCV13 serotypes, PCV15 contains 2 additional serotypes (22F and 33F), while PCV20 contains 7 additional serotypes (8, 10A, 11A, 12F, 15B, 22F and 33F), that are among the most prevalent serotypes causing pneumococcal disease in adults. The suggested utilization of these newer conjugate vaccines was based on immunogenicity and safety studies (17–22).

The Greek NIP was recently modified considering the higher valency PCVs. According to the latest update, as provided by the Greek Ministry of Health on 07 February 2023, all pneumococcal vaccine-naïve persons ≥65 years of age and persons 18–64 years of age at increased risk for pneumococcal disease are suggested to receive 1 dose of PCV20. There are also specific recommendations for vaccination with PCV20 in adults with prior pneumococcal vaccination who are considered as not having completed their pneumococcal vaccination series (23).

Understanding the economic implications of updates in preventive and therapeutic public healthcare protocols is crucial for aiding healthcare decision-makers in the prudent utilization of limited healthcare resources, particularly in countries where pricing and reimbursement determinations rely on scientific proof through health technology assessment. In this light, the aim of the current study was to evaluate the cost-effectiveness of PCV20 compared to PCV15 alone or followed by PPV23 for adults in Greece.

## 2. Materials and methods

### 2.1. Model structure overview

**Figure 1:**
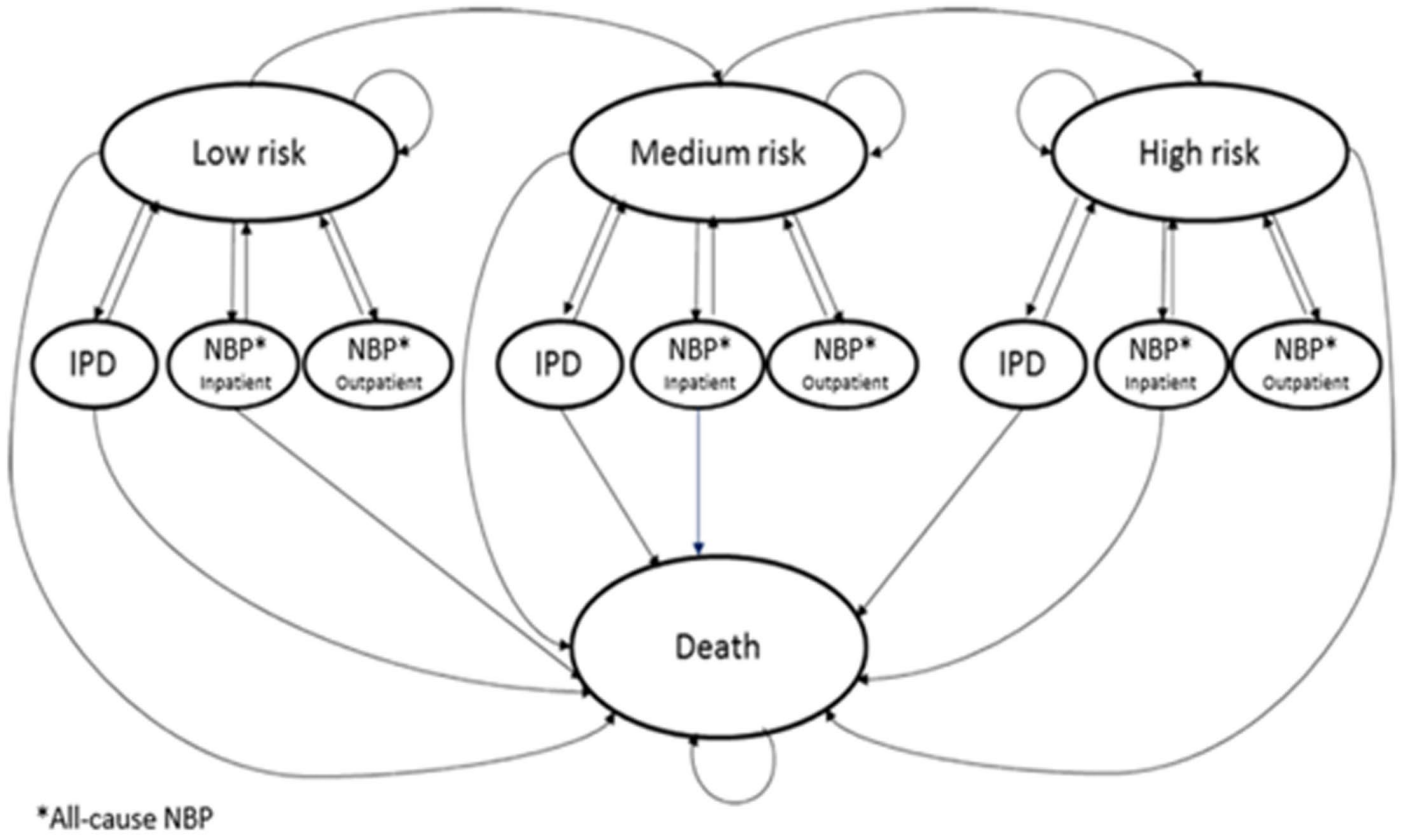
Model structure.

A published deterministic model (24) with a Markov-type process was used to depict the lifetime risk of IPD and all-cause non-bacteremic pneumonia (NBP) and associated costs in adults aged 18–64 years with underlying conditions and all adults aged 65 years and older in Greece. Upon entering the model, the population is categorized based on age and risk profile, which includes low, moderate, or high risk. Persons may transition to a higher risk group, but not to a lower risk group, during the modeling lifetime horizon (82 years) (Figure 1).

Annually, the projected clinical outcomes and economic costs for the model population are determined, taking into account various factors such as age, risk profile, disease/fatality rates, vaccination status, as well as the costs associated with vaccinations and medical care. The incidence of IPD is inclusive of both bacteremia and meningitis, while the occurrence of non-NBP is categorized by the care setting (inpatient or outpatient) for all causes. Individuals who have received the vaccine may experience a reduced risk of future IPD and all-cause NBP. The extent of risk reduction associated with vaccination depends on factors such as clinical presentation, types of vaccines used, the proportion of preventable diseases, as well as age and risk profile. Risk of death from IPD, all-cause NBP, and other causes is contingent upon age and individual risk profile. Expected medical treatment costs for IPD and all-cause NBP are generated based on event rates and unit costs in relation to care setting. The cost related to vaccination are accounted for in the year when the vaccine is administered. For each vaccination strategy, clinical outcomes and economic costs projected, including IPD and all-cause NBP cases and attributable deaths, life-years (LYs), quality-adjusted LYs (QALYs), and costs of vaccination and medical care treatment. The analysis was conducted from the perspective of a Greek public payer (EOPYY), and future model outcomes were discounted annually at a rate of 3.5%.as often used in such studies (25–27).

### 2.2. Model population and comparators

The model population size assessed in the cost-effectiveness model was Greek adults eligible for pneumococcal vaccination, divided in 5 age-groups (18–49 years, 50–64 years, 65–74 years, 75–84 years, 85–99 years) extracted from National Statistical Authority (28). The distributions of persons in each age group were allocated into low-, moderate and high-risk subgroup according to the existence of chronic and underlying comorbidities, based on local clinical experts’ opinion (Table 1). Meanwhile, the persons in the model population were assumed to receive either PCV20 alone, PCV15 alone or PCV15 followed by PPV23 during the modeling horizon.

**Table 1:** Health inputs considered in the model.

Age and risk profile																Source
	18–49 years (*n* = 4,606,725)			50–64 years (*n* = 2,198,121)			65–74 years (*n* = 1,177,281)			75–84 years (*n* = 831,503)			85–99 years (*n* = 377,416)			(28)
	Low risk	*Moderate risk*	High risk	Low risk	Moderate risk	High risk	Low risk	Moderate risk	High risk	Low risk	Moderate risk	High risk	Low risk	Moderate risk	High risk	
*Risk distribution, % of n*	68.8%	20.6%	10.6%	47.2%	33.9%	18.9%	29.4%	43.1%	27.5%	16.1%	55.0%	28.9%	6.9%	53.5%	39.6%	*Local experts*
*Annual disease incidence (per 100,000)*																
*IPD*																
*Bacteremia*	2.5	8.4	24.0	6.2	21.0	60.0	8.6	24.0	37.7	10.2	28.5	44.7	14.8	41.5	65.2	*Local experts*
*Meningitis*	0.2	0.7	1.9	0.5	1.6	4.7	0.7	1.9	2.9	0.8	2.2	3.5	1.2	3.2	5.1	
*NBP*																
*Hospitalized*	69	249	467	199	720	1,349	471	1,424	1,910	722	2,183	2,928	1,192	3,601	4,830	*Local experts*
*Outpatient Care*	187	676	1,266	236	851	1,594	523	1,579	2,144	805	2,434	3,303	1,121	3,338	4,598	
*Annual mortality/case-fatality (per 100)*																
*IPD*																
*Bacteremia*	*6*	*8*	*10*	*11*	*12*	*13*	*11*	*14*	*14*	*11*	*14*	*14*	*19*	*20*	*24*	*Local experts*
*Meningitis*	*6*	*8*	*10*	*11*	*12*	*13*	*11*	*14*	*14*	*11*	*14*	*14*	*19*	*20*	*24*	
*NBP*																
*Hospitalized*	*0.6*	*0.8*	*2.6*	*1*	*2.5*	*5.4*	*3.1*	*4.6*	*6.8*	*6.8*	*8.4*	*9*	*8.2*	*8.4*	*11*	*Local experts*
*Outpatient Care*	*0*	*0*	*0*	*0*	*0*	*0*	*0*	*0*	*0*	*0*	*0*	*0*	*0*	*0*	*0*	
*Vaccine effectiveness PCV* vs. *VT-IPD*																
*Year 1*	*81.5%*	*81.5%*	*65.2%*	*79.2%*	*79.2%*	*63.3%*	*75%*	*75%*	*60%*	*75%*	*75%*	*60%*	*75%*	*75%*	*60%*	(29–32)
*Year 5*	*81.5%*	*81.5%*	*65.2%*	*79.2%*	*79.2%*	*63.3%*	*75%*	*75%*	*60%*	*75%*	*75%*	*60%*	*75%*	*75%*	*60%*	
*Year 10*	*63.1%*	*63.1%*	*50.5%*	*61.2%*	*61.2%*	*49%*	*58%*	*58%*	*46.4%*	*58%*	*58%*	*46.4%*	*58%*	*58%*	*46.4%*	
*Year 15*	*37.2%*	*37.2%*	*29.8%*	*36.2%*	*36.2%*	*28.9%*	*34.3%*	*34.3%*	*27.45*	*34.3%*	*34.3%*	*27.45*	*34.3%*	*34.3%*	*27.45*	
*Year 16+*	*0%*	*0%*	*0%*	*0%*	*0%*	*0%*	*0%*	*0%*	*0%*	*0%*	*0%*	*0%*	*0%*	*0%*	*0%*	Assumption
*Vaccine effectiveness PCV* vs. *VT-NBP*																
*Year 1*	*55.6%*	*55.6%*	*44.5%*	*51.3%*	*51.3%*	*41.1%*	*45%*	*45%*	*36%*	*45%*	*45%*	*36%*	*45%*	*45%*	*36%*	(29–32)
*Year 5*	*55.6%*	*55.6%*	*44.5%*	*51.3%*	*51.3%*	*41.1%*	*45%*	*45%*	*36%*	*45%*	*45%*	*36%*	*45%*	*45%*	*36%*	
*Year 10*	*43%*	*43%*	*34.3%*	*39.7%*	*39.7%*	*31.8%*	*34.8%*	*34.8%*	*27.9%*	*34.8%*	*34.8%*	*27.9%*	*34.8%*	*34.8%*	*27.9%*	
*Year 15*	*25.4%*	*25.4%*	*20.3%*	*23.5%*	*23.5%*	*18.8%*	*20.6%*	*20.6%*	*16.4%*	*20.6%*	*20.6%*	*16.4%*	*20.6%*	*20.6%*	*16.4%*	
*Year 16+*	*0%*	*0%*	*0%*	*0%*	*0%*	*0%*	*0%*	*0%*	*0%*	*0%*	*0%*	*0%*	*0%*	*0%*	*0%*	Assumption
*Vaccine effectiveness* PPV23 vs. VT-IPD																
*Year 1*	*59.1%*	*32.8%*	*17.1%*	*58.3%*	*32.3%*	*16.8%*	*55.7%*	*30.9%*	*16.1%*	*50.8%*	*28.1%*	*14.6%*	*37.9%*	*20.5%*	*10.6%*	(33)
*Year 5*	*45.1%*	*25%*	*13%*	*44.4%*	*24.6%*	*12.8%*	*42.5%*	*23.5%*	*12.2%*	*38.7%*	*21.4%*	*11.1%*	*28.9%*	*15.6%*	*8.1%*	
*Year 10+*	*0%*	*0%*	*0%*	*0%*	*0%*	*0%*	*0%*	*0%*	*0%*	*0%*	*0%*	*0%*	*0%*	*0%*	*0%*	Assumption

IPD, invasive pneumococcal disease; NBP, non-bacteremic pneumonia; PCV, pneumococcal conjugate vaccine; PPV23, 23-valent pneumococcal polysaccharide vaccine; VT, vaccine-type.

### 2.3. Model parameters

**Figure 2:**
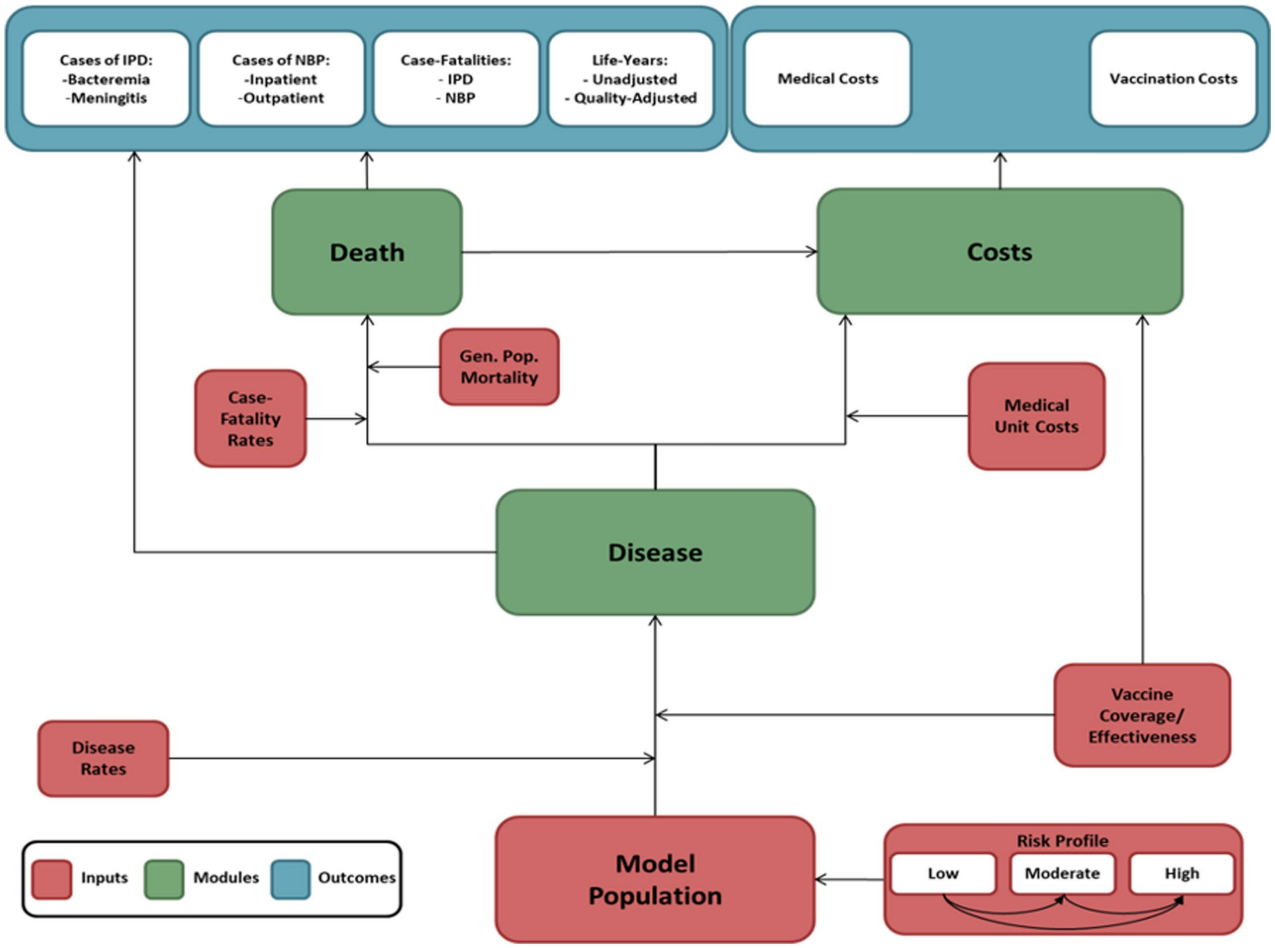
A schematic of the model inputs and the outcomes.

The model inputs considered epidemiology, utilities, vaccine serotype coverage, vaccine effectiveness and uptake, and direct medical costs. These data which are presented in detail in the following sections were obtained from published studies and official Greek sources. The literature review and data collection were performed up to March 2023. A schematic of the model inputs and the outcomes considered is presented in Figure 2.

#### 2.3.1. Disease incidence and mortality

The disease incidence considered in the model was divided into bacteremia, meningitis and all-cause NBP (inpatient or outpatient care setting) incidence. Annual incidence of bacteremia, meningitis, and all-cause NBP were estimated by age and risk profile and were based on local clinical experts’ opinion (Table 1).

Mortality data were also divided into bacteremia, meningitis and all-cause NBP (inpatient or outpatient care setting) fatality and estimated by age and risk profile (Table 1). The mortality data of bacteremia, meningitis and all-cause NBP were derived from published studies and validated by local clinical experts, while the general population mortality was obtained from WHO National Life Tables for Greece (latest available).

#### 2.3.2. Vaccine coverage and efficacy

The vaccine effectiveness (VE) of PCVs against VT-IPD and VT-NBP, for low−/moderate-risk persons aged 50–64 years, was derived using age-specific relative changes in VE against VT-IPD and VT-NBP (vs. age 65 years) from Mangen et al. (29). For individuals aged 18–49 years, the VE was assumed to be the same as that for individuals aged 50 years (29). For low−/moderate-risk individuals aged ≥65 years, VE obtained from randomized controlled trial data from the Community-Acquired Pneumonia Immunization Trial in Adults (CAPiTA) (29). For high-risk individuals VE was assumed to be equal to 80% of corresponding values for low−/moderate-risk persons (Table 1). This assumption was supported by findings from literature (30, 31) and a recent published cost-effectiveness study of PCV20 in the UK (24). Initial VE of PCVs was assumed to persist for 5 years, consistent with the CAPiTA trial (29, 32) and to annually wane thereafter, 5% during years 6–10 and 10% between years 11 and 15. After year 16, it was assumed that there was no efficacy through the end of the model horizon. These assumptions were also used in similar published cost-effectiveness studies (24, 34) (Table 1).

The VE of PPV23 against VT-IPD for low-, moderate-, and high-risk persons was derived for all ages by fitting a logarithmic curve to values for persons aged 65–74, 75–84, and 85–99 years, and then estimating the age-specific values across the three risk groups using relative risks from Djennad et al. and the population sizes (33) (Table 1). Based on published studies, it was assumed that the VE of PPV23 against VT-NBP was zero (35, 36) and consistent with base-case assumptions employed in several of economic evaluation studies (24, 34, 37–39). As for the vaccine waning for PPV23, it was obtained from Djennad et al. (33), with a linear decline to 76.2% of initial vaccine efficacy by year 5, followed by a linear decline to no efficacy by year 10. This assumption was also used in similar published cost-effectiveness studies (24, 34) (Table 1).

Age and risk profile specific vaccine uptake was considered in the analysis, and was assumed to be higher for persons aged 65 years and older compared to persons aged <64 years, based on local clinical experts. Vaccine uptake was assumed to be the same for all vaccination strategies. Moreover, vaccine serotype coverage was also included in the model, however in absence of detailed local data, the proportion of IPD and NBP against which the vaccines provide protection were derived from published data (40) and validated by local clinical experts (41). The assumption was made that the coverage against serotypes would be consistent across all age groups.

#### 2.3.3. Utilities data

Age- and risk-specific health-state utility values for the general population were derived from published study (42). Moreover, the annual disutility associated with hospitalized disease (i.e., bacteremia, meningitis, and all-cause NBP) was −0.13 and based on a study by Mangen et al. (43). Annual disutility of NBP requiring outpatient care only (−0.004) was based on data from a study by Melegaro et al. (44). Regardless of age and risk profile, it was assumed that all disutilities were equal (Table 2).

**Table 2:** Health-state utility and disutility inputs considered in the model.

Age (years)/Risk group	Health-State Utility (42)	Disutility			
		IPD		All-Cause NBP	
		Bacteremia (43)	Meningitis (43)	Hospitalized (43)	Outpatient Care (44)
18–49 years					
Low	0.8998	−0.13	−0.13	−0.13	−0.004
Moderate	0.8998	−0.13	−0.13	−0.13	−0.004
High	0.8247	−0.13	−0.13	−0.13	−0.004
50–64 years					
Low	0.8084	−0.13	−0.13	−0.13	−0.004
Moderate	0.8084	−0.13	−0.13	−0.13	−0.004
High	0.6870	−0.13	−0.13	−0.13	−0.004
65–74 years					
Low	0.7658	−0.13	−0.13	−0.13	−0.004
Moderate	0.7658	−0.13	−0.13	−0.13	−0.004
High	0.6049	−0.13	−0.13	−0.13	−0.004
75–84 years					
Low	0.7183	−0.13	−0.13	−0.13	−0.004
Moderate	0.7183	−0.13	−0.13	−0.13	−0.004
High	0.5423	−0.13	−0.13	−0.13	−0.004
85–99 years					
Low	0.5392	−0.13	−0.13	−0.13	−0.004
Moderate	0.5392	−0.13	−0.13	−0.13	−0.004
High	0.5102	−0.13	−0.13	−0.13	−0.004

IPD, invasive pneumococcal disease; NBP, non-bacteremic pneumonia.

#### 2.3.4. Costing data

The analysis was conducted from a public payer perspective and as such only health care costs reimbursed by the public payer were considered. In particular, the cost inputs considered in the model included vaccination costs and medical treatment costs for IPD and all-cause NBP. All unit costs correspond to the year of the analysis (2022, €).

In Greece, vaccines are fully reimbursed from the public payer, as per current legislation. The vaccine unit costs were sourced from the latest available price bulletin issued by the Greek Ministry of Health as well as the official list of the reimbursed medicines (Table 3) (45).

**Table 3:** Cost inputs considered in the model.

	List price	Source
*Vaccine cost*		
20-valent pneumococcal conjugate vaccine	€ 70.97	Price Bulletin issued by the Greek Ministry of Health (45)
23-valent pneumococcal polysaccharide vaccine	€ 31.45	
15-valent pneumococcal conjugate vaccine	€ 72.32	

Medical care costs^*^						
Age (years)	*18–64 years*			*65–99 years*		
Profile risk	Low	Moderate	High	Low	Moderate	High
*IPD*						
*Bacteremia*	€2,316	€2,460	€2,820	€2,316	€2,604	€2,892
*Meningitis*	€1,579	€2,370	€2,658	€2,226	€2,442	€2,730
*NBP*						
*Hospitalized*	€229	€645	€2,122	€547	€1,048	€2,338
*Outpatient Care*	€14	€12	€0^¥^	€13	€9	€0^¥^

IPD, invasive pneumococcal disease; NBP, non-bacteremic pneumonia.

* Diagnosis-Related Group tariffs (39), Price Bulletin (45), official website of EOPYY (46) and Local experts.

¥ Based on local experts, it was assumed that all patients at high risk required hospitalization.

As for the management cost of bacteremia, meningitis and all-cause non-bacteremic pneumonia, this was derived after investigating the setting on which it is treated. More specifically, all patients with bacteremia and meningitis were considered as requiring hospitalization. For the case of pneumonia, local experts provided the proportion of patients deemed in need of hospitalization. Moreover, costs for treating and managing events in the inpatient setting were obtained from the corresponding Diagnosis-Related Group (DRGs) tariffs issued by the Greek Ministry of Health (47). Combining the resources utilized, the costs associated with outpatient management of pneumonia were estimated as provided by local experts, with the corresponding unit costs obtained from the drug price bulletin issued by the Greek Ministry of Health (45), Government gazette and the official website of public payer (EOPYY) (46) (Table 3).

## 3. Analysis

Using the aforementioned method and data, the calculation was performed to determine the clinical outcomes and economic costs for each vaccination strategy. The main model outcomes included IPD and all-cause NBP cases and attributable deaths, LYs and QALYs gained, and lifetime costs. An incremental cost-effectiveness analysis was performed to identify the most cost-effective strategy. Despite the fact that, there is no official willingness-to-pay (WTP) threshold for Greece for a health intervention to be considered cost-effective, a WTP threshold of €34,000 per QALY/LY was used in the current analysis based on the recommendation that a health intervention should be considered cost-effective if the ICER is between one to three times the GDP *per capita* of that country (48–51). This formula for WTP threshold has been widely used in cost-effectiveness studies within global health (48–51). The Greek GDP *per capita* was taken from the International Monetary Fund (IMF), which estimated it at €17,000 using current prices (52) at the time of the analysis.

A one-way sensitivity analysis (OWSA) was undertaken to test the robustness of the results to individual inputs, holding all else constant. The parameters considered in OWSA were disease incidences, mortality, utility, disutility, VE, medical cost, and vaccine price. OWSA’s model results were recorded after changing each input to its upper and lower bound value in turn. The upper and lower bound values for each parameter were taken as percent of base case (−25% for lower bound and + 25% for upper bound).

Moreover, probabilistic sensitivity analysis (PSA), which assesses the stochastic parametric uncertainty, was used. This is a technique that provides an estimation of the joint uncertainty of costs and effectiveness, based on a simulation where assigning probabilistic distributions to key input parameters, recursively re-sampling new values for each parameter from their respective distribution, and subsequently estimating the costs and effectiveness of each intervention based on the new values is undertaken. A normal distribution was applied for disease incidences and mortality. A beta distribution was used for estimates of utility and disutility, while a triangular distribution was applied for VE. The PSA used simulation modeling to run 1,000 analyses, in order to be able to construct cost-effectiveness acceptability curves (CEAC), which indicate the likelihood of the incremental cost per QALY to fall below specified thresholds.

In addition, scenario analyses were conducted to assess the cost-effectiveness of vaccination of specific age/risk subgroups. More specifically, different age and risk profile groups such as (i) individuals aged 65 years and older at moderate or high risk, (ii) individuals aged 65 years and older at high risk, (iii) individuals aged 18–64 years at high risk and (iv) individuals aged 65 years and older were considered.

## 4. Results

### 4.1. Base case results

According to the base case analysis PCV20 resulted in fewer IPD and NBP cases and deaths compared to either PCV15 alone or PCV15 followed by PPV23. More specifically, over the modeled time horizon, vaccination with PCV20 compared to PCV15 alone or PCV15 followed by PPV23 prevents an additional 747 and 646 cases of IPD, 10,334 and 10,342 cases of NBP and 468 and 455 deaths respectively, resulting in incremental gain of 1,594 and 1,536 QALYs, respectively, (Table 4).

**Table 4:** Base case model results.

Parameters	PCV15 ➔ PPV23	PCV15 alone	PCV20	Differences	
				PCV20 *vs* PCV15 ➔ PPV23	PCV20 *vs* PCV15 alone
*Health outcomes*					
IPD (No. of cases)	63,734	63,835	63,088	−646	−747
Bacteremia	59,107	59,201	58,508	−599	−693
Meningitis	4,627	4,634	4,580	−47	−54
All-Cause NBP (No. of cases)	7,022,256	7,022,248	7,011,914	−10,342	−10,334
Inpatient	3,320,621	3,320,617	3,315,683	−4,938	−4,934
Outpatient	3,701,635	3,701,631	3,696,231	−5,403	−5,399
No. of deaths	241,061	241,074	240,605	−455	−468
Total Quality-Adjusted Life Years	92,501,752	92,501,694	92,503,288	1,536	1,594
Total life years	119,539,860	119,539,781	119,541,743	1,883	1,962
*Economic outcomes*					
Medical care and vaccination cost	€ 2,545,278	€ 2,507,603	€ 2,496,420	-€ 48,858	-€ 11,183
*Incremental cost-effectiveness ratio (ICER)*					
Cost per QALY	–		–	Dominant	Dominant
Cost per LY	–		–	Dominant	Dominant

IPD, invasive pneumococcal disease; NBP, non-bacteremic pneumonia; PCV15, 15-valent pneumococcal conjugate vaccine; PCV20, 20-valent pneumococcal conjugate vaccine; PPV23, 23-valent pneumococcal polysaccharide vaccine; LYs, Life Years; QALYs, Quality Adjusted Life Years.

The lower number of pneumococcal disease cases with PCV20 compared to PCV15 alone or PCV15 followed by PPV23 translated to a reduction of medical costs of €11,183 and €48,858 respectively, over the model life-time horizon (Table 4).

The incremental analysis showed that, PCV20 compared to PCV15 alone or PCV15 followed by PPV23 resulted in both a QALY gain and a cost reduction. Hence, based on these findings, vaccination with PCV20 was estimated to be a dominant strategy (improved health outcomes with reduced costs) compared with PCV15 alone or PCV15 followed by PPV23 (Table 4).

### 4.2. Sensitivity and scenario analyses results

The OWSA demonstrated the resilience of the findings to variations in the base case parameters of the model. It is important to mention that in all sensitivity analyses, vaccination with PCV20 was associated with lower costs and more QALYs gained as compared to PCV15 alone or PCV15 followed by PPV23, hence, PCV20 remained a dominant vaccination strategy. Moreover, the PSA confirmed the base case results. In particular, the analyses showed that at the predefined WTP of €34,000 per QALY/LY gained, vaccination with PCV20 had 100% probability of being a cost-effective option compared with PCV15 alone or PCV15 followed by PPV23.

Scenario analyses according to specific age- and risk- groups were also conducted. In all scenario analyses, PCV20 remains a cost-effective option compared with PCV15 alone or PCV15 followed by PPV23 (Supplementary file, Table 1).

## 5. Discussion

Following recent approval of PCVs with expanded serotype coverage, the present study was undertaken from a public payer perspective to compare the health and economic outcomes of PCV20 with those of PCV15 vaccination strategies for adults in Greece. PCV20 was estimated to be a dominant vaccination strategy (improved health outcomes with reduced costs) over PCV15 alone or PCV15 followed by PPV23 for prevention of pneumococcal disease in adults in Greece.

The results of sensitivity analyses revealed that the base-case findings were not significantly affected by changes in input parameters and assumptions. In particular, PSA estimated that vaccination with PCV20 had a 100% probability of being a cost-effective vaccination option compared to PCV15 alone or followed by PPV23, respectively, under the WTP threshold of €34,000 per QALY gained. Furthermore, the OWSA revealed that in all sensitivity analyses, vaccination with PCV20 was associated with lower costs and more QALYs gained as compared to PCV15 alone or PCV15 followed by PPV23.

At the time of writing, a limited number of economic studies have been conducted to assess the cost-effectiveness of PCV20 compared to PCV15 or PCV15 followed by PPV23 were found in the international literature, however all of them are aligned with our results (53–55). More specifically, a cost-effectiveness study (54) conducted in Argentina from a payer’s perspective revealed that PCV20 compared to PCV15 followed by PPV23 averted more cases of IPD, all-cause NBP and deaths with a higher number of LYs and QALYs at a lower cost, hence, PCV20 was estimated to be a dominant vaccination strategy. Moreover, a recent study (53) conducted in Italy showed that PCV20 was a cost-effective vaccination strategy versus PCV15 alone (assuming a price that is equivalent to that of PCV13) in adults’ population. Additionally, a study performed in USA showed that PCV20 use are substantially more economically reasonable in the older adult population than PCV15 followed by PPSV23 (55).

The cost-effectiveness results stem from the inclusion of additional serotypes in PCV20 compared to PCV15 (8, 10A, 11A, 12F, and 15B) and PCV13 (8, 10A, 11A, 12F, 15B, 22F, and 33F), which are major causes of pneumococcal disease in the adult population. Despite the lack of a comprehensive national surveillance system for pneumococcal disease in Greece, that precluded the use of local morbidity and epidemiological data in our cost-effectiveness model, there are some recently published data underlining the clinical value of PCV20 in Greece (8, 41). According to the National Meningitis Reference Laboratory data for the period 2010–2020, in adults aged ≥65 years, PCV13, PCV15 and PCV20 were estimated to potentially cover 41.1, 42.5 and 54.8% of pneumococcal meningitis cases, respectively (8). In addition, in the interim analysis of an ongoing prospective study of adults ≥19 years old hospitalized with clinical and radiographically-confirmed community-acquired pneumonia (CAP) in the Ioannina and Kavala regions of Greece (EGNATIA study), PCV13, PCV15 and PCV20 were estimated to potentially cover 7.3, 8.1 and 9.8% of hospitalized all-cause CAP in adults aged ≥19 years, respectively (41).

Moreover, Greece, like many other countries, has an aging population. Older adults are particularly vulnerable to pneumococcal infections, as immunosenescence renders them more susceptible to disease and severe complications. According to a new demographic study, by 2,100 the proportion of the population aged 65 years and older is projected to increase, reaching one third of the overall Greek population (56). Therefore, it is of utmost importance to protect this high-risk population with appropriate and effective vaccines offering broad serotype coverage.

Furthermore, the additional serotypes of PCV20 compared to PCV13 are important in the epidemiology of pneumococcal disease not only in adults, but also in the pediatric population. In Greece, according to the National Meningitis Reference Laboratory pneumococcal meningitis data for the period 2010–2020, PCV13 serotypes represented 27.3%, PCV15 serotypes also represented 27.3%, and PCV20 serotypes represented 47.7% of pneumococcal meningitis cases in children aged <5 years (8). Moreover, another study which was conducted in Greece from November 2015–December 2020 in children aged ≤14 years with IPD and non-invasive pneumococcal disease (mainly otitis media) reported that PCV13 serotypes were responsible for 42.9% of pneumococcal disease (38.8% for IPD, 44.7% for non-invasive pneumococcal disease). PCV15 and PCV20 were estimated to potentially cover 46.3% (42.9% of IPD, 47.7% of non-invasive pneumococcal disease) and 64.9% (66.3% of IPD, 64.3% of non-invasive pneumococcal disease) of pneumococcal disease in children aged ≤14 years (57), respectively. Finally, in a cross-sectional study using molecular methods, data on oropharyngeal pneumococcal colonization over time was collected from 1,212 Greek children throughout the country during the period January–August 2017. Serotypes/serogroups 15A/B/C/F, 11A/D/E, 10A/B, 22A/F, 33F, 12A/B/F/44/46, 8, were all among the identified serotypes/serogroups in pneumococcal carriage (58).

Lastly, while cost-effectiveness analysis is a useful tool for evaluating the costs and benefits of health interventions, it typically does not capture all the value elements that vaccines offer. More specifically, vaccination can also have wider societal benefits, such as reducing the burden on healthcare systems, improving productivity by reducing absenteeism from work and school, helping combat antimicrobial resistance and preventing outbreaks that can have significant economic impacts (59, 60). These benefits are not always easy to quantify and may not be captured by a cost-effectiveness analysis, and that is true of the current analysis. In addition, the COVID-19 pandemic has highlighted the importance of vaccination in reducing the transmission of the virus, protecting populations from severe illness and death and supporting the society’s return to normality (59, 60). In this context, the value of vaccination may extend beyond the traditional cost-effectiveness analysis framework and a more comprehensive approach is needed to fully capture the societal and economic benefits of vaccination and to ensure that the health policy decisions makers are aligned with the broader goals of public health.

In terms of our study limitations, in the current analysis, it was postulated that the utility and disutility data acquired from the published studies (42–44) were relevant to the healthcare environment in Greece, nonetheless, considering the lack of available data at the local level and the limitations in terms of quality and validity of the relevant studies, this decision was deemed suitable. Moreover, whenever necessary, local experts were consulted and their input, including any pertinent local data, was taken into account to ensure the validation of the model’s inputs. However, a set of sensitivity analyses demonstrated the robustness of the model outcomes, as the key findings remained consistent across a broad spectrum of parameter values. Furthermore, the present model analysis did not include potential adverse events (AE) associated with PCV20, PCV15 and PPV23 vaccination, since most AE are of mild or moderate severity and serious AE are rare, hence the impact on model base case results would be negligible. The same approach has been used also in recent model publications in UK and Denmark (24, 34). However, in the absence of differential safety data, incorporating AEs would favor single-dose strategies. Additionally, the present analysis was undertaken from the viewpoint of the public payer perspective, focusing solely on direct costs. While adopting a societal perspective could prove valuable, the current analysis did not incorporate indirect costs, such as patient time, caregiver expenses, and productivity losses, which would reflect the missed opportunities for society as a whole. Moreover, it should be noted that the results have to be considered for the management of pneumococcal disease in the strict Greek setting and on the basis of the present time, disease costs, vaccine prices, epidemiology data and the NIP. Therefore, the results of current study may not be generalizable to other populations with different disease burdens and healthcare systems.

## 6. Conclusion

PCV20 is estimated to improve public health by averting additional pneumococcal disease cases and deaths relative to PCV15 alone or PCV15 followed by PPV23, and translates to cost-savings for the public payer. Overall results showed that vaccination with PCV20 was estimated to be a dominant vaccination strategy (improved health outcomes with reduced costs) over PCV15 alone or PCV15 followed by PPV23 for prevention of pneumococcal disease in adults.

## Data availability statement

The original contributions presented in the study are included in the article/Supplementary material, further inquiries can be directed to the corresponding author.

## Ethics statement

This study was an economic evaluation analysis which based on previously publicly available data and does not involve any new studies of human or animal subjects performed by any of the authors.

## Author contributions

GG and CT conducted the analyses, collected the data, and interpreted the results. GG, MB, and VK wrote the manuscript. GG, MB, VK, JV, and CT contributed to results interpretation and manuscript writing. All authors reviewed and approved the final manuscript.
