# Supplementary material for: Health and economic outcomes of 20-valent pneumococcal conjugate vaccine compared to 15-valent pneumococcal conjugate vaccine strategies for adults in Greece
Source: Front Public Health. 2023 Sep 29;11:1229524. doi: 10.3389/fpubh.2023.1229524 (PMC10570410; doi:10.3389/fpubh.2023.1229524)
Supplement: Supplementary file 1 [file Data_Sheet_1.PDF]

Supplementary File

Table 1: Scenario analyses results (*per person model results)*

**Parameters**	**PCV20 vs PCV15 alone**	**PCV20 vs PCV15 → PPV23**
1. All persons from 65 years and older		
Incremental Costs	-€3	-€17
Incremental QALYs	0.0006	0.0006
Incremental LYs	0.0008	0.0008
**ICER per QALY gained**	**Dominant**	**Dominant**
**ICER per LY gained**	**Dominant**	**Dominant**
2. Moderate and high-risk persons from 65 years and older		
Incremental Costs	-€4	-€17
Incremental QALYs	0.0007	0.0007
Incremental LYs	0.0009	0.0009
**ICER per QALY gained**	**Dominant**	**Dominant**
**ICER per LY gained**	**Dominant**	**Dominant**
3.High-risk persons from 65 years and older		
Incremental Costs	-€5	-€19
Incremental QALYs	0.0008	0.0007
Incremental LYs	0.0008	0.0010
**ICER per QALY gained**	**Dominant**	**Dominant**
**ICER per LY gained**	**Dominant**	**Dominant**
4. High-risk persons 18-64 years old		
Incremental Costs	-€2	-€5
Incremental QALYs	0.0005	0.0005
Incremental LYs	0.0006	0.0006
**ICER per QALY gained**	**Dominant**	**Dominant**
**ICER per LY gained**	**Dominant**	**Dominant**

Abbreviations: ICER, Incremental Cost-Effectiveness Ratio; LYs, Life Years; PCV20, 20-valent pneumococcal conjugate vaccine; PCV15, 15-valent pneumococcal conjugate vaccine; PPV23, 23-valent pneumococcal polysaccharide vaccine; QALYs, Quality Adjusted Life Years.
